# Not every sperm counts: Male fertility in solitary bees, *Osmia cornuta*

**DOI:** 10.1371/journal.pone.0214597

**Published:** 2019-03-28

**Authors:** Verena Strobl, Lars Straub, Selina Bruckner, Matthias Albrecht, Jakkrawut Maitip, Eleonora Kolari, Panuwan Chantawannakul, Geoffrey R. Williams, Peter Neumann

**Affiliations:** 1 Institute of Bee Health, Vetsuisse Faculty, University of Bern, Bern, Switzerland; 2 Swiss Bee Research Centre, Agroscope, Bern, Switzerland; 3 Department of Entomology & Plant Pathology, Auburn University, Auburn, AL, United States of America; 4 Agroecology and Environment, Agroscope, Zürich, Switzerland; 5 Faculty of Science, Energy and Environment, King Mongkut's University of Technology, North Bangkok, Rayong Campus, Bankhai, Rayong, Thailand; 6 Bee Protection Center, Department of Biology, Faculty of Science, Chiang Mai University, Chiang Mai, Thailand; 7 Environmental Science Research Center, Faculty of Science, Chiang Mai University, Chiang Mai, Thailand; University of Missouri Columbia, UNITED STATES

## Abstract

Reproductive strategies can act as strong selective forces on reproductive traits of male insects, resulting in species-specific variation in sperm quantity and viability. For solitary bees, basic measures of sperm quantity and viability are scarce. Here we evaluated for the first time quantity and viability of sperm in male *Osmia cornuta* solitary bees at different times after emergence, and how they were affected by male body mass and environmental condition (laboratory or semi-field arena). Sperm viability immediately after adult emergence showed no significant difference compared to four day old individuals, suggesting that *O*. *cornuta* males are capable of mating immediately post emergence. However, sperm counts were significantly higher in four day old individuals from the semi-field arena when compared to newly emerged males. This might reflect a final phase of sperm maturation. Regardless of individual male age and body mass differences, *O*. *cornuta* males produced on average ~175’000 spermatozoa that were ~65% viable, which are both significantly lower compared to eusocial honeybees and bumblebees. Moreover, sperm quantity, but not viability, was positively correlated with male body mass four days after emergence, while no such relationship was detected immediately after emergence. Even though individuals maintained in semi-field conditions exhibited a significantly greater loss of body mass, experimental arena had no significant effect on male survival, sperm quality or total living sperm produced. This suggests that the proposed laboratory design provides a cost-efficient and simple experimental approach to assess sperm traits in solitary bees. In conclusion, our data suggest a reduced investment in both sperm quantity and quality by male *O*. *cornuta*, which appears to be adaptive in light of the life history of this solitary bee.

## Introduction

Numerous examples exist in nature of males adapting to promote their reproductive success. For example, a range of post-copulatory behavioral traits of male insects prevent females from additional mating, while morphological adaptations exist to ensure the displacement or removal of rival sperm from the site of fertilization [1,2]. Such traits belong to the most rapidly evolving characters [3], whereby sperm competition is argued to be a central force [4,5]. Sperm size, length, quantity and viability are a few characteristics of the male ejaculate that can considerably vary depending on post-copulatory sexual selection [6,7]. Further factors that govern variability in male sperm traits can include species-specific life histories and behavioral aspects such as mating strategies [8,9].

Mating strategies in insects range from monogamy to polygamy [10]. These strategies are often intimately linked to other reproductive parameters [11] such as duration of copulation [12], courtship behavior, and sperm traits [13,14]. Polyandry, wherein females mate with multiple males, occurs in many insects [15], and has been studied in detail in the eusocial Hymenoptera (e.g. ants, social bees and wasp species) [16]. In the case of the honeybee, queens are known to mate with multiple males (drones) [17], which inevitably die post copulation [18]. Consequently, honeybee drones only have a single chance to ensure paternity and therefore produce high sperm numbers with extremely high viability to enhance fertilization changes during post-copulatory sperm competition over female’s ova [19–22]. It has been argued that the most critical effect of polyandry on male individuals arises because of sperm competition and cryptic female choice, with polyandry favoring increased male ejaculate expenditure [2]. Additionally, in large drone congregation areas with extremely male biased sex ratios, males have to compete with several thousands of rivals for copulation [23]. Thus body size is an important trait because larger drones have higher mating chances [24] and sperm numbers are positively correlated with body size [19].

Not all social bee species display polyandry. For instance, most bumblebee (*Bombus*) and stingless bee species display monandry [25,26]. Despite the lack of post-copulatory sperm competition in *Bombus terrestris* due to monandry, as well as male survival post-copulation, sperm viability values in this species are similar to those observed in honeybees [20,27,28]. This is most likely due to females relying on large sperm numbers to successfully establish sufficiently large colonies.

Despite a lack of behavioral observations and genetic pedigree analysis, females of most solitary bee species are believed to display monandry [29]. In monandrous mating systems, males can only increase their fitness by inseminating several females [30]. Therefore, males that encounter receptive virgin females first are likely to have a reproductive advantage [31]. Sexual selection should therefore favor males that are able to locate a female quickly [10], rapidly discriminate between receptive and non-receptive females [32], and successfully defend their territories against rival males [33]. However, for solitary bees few data exist on sperm traits of males and how this may play a role in reproductive strategies of species [15,34,35]. In Hymenoptera, females also have control over their offspring sex with fertilized eggs usually developing into females and non-fertilized ones into males [36]. Therefore, male hymenopteran fitness depends on female offspring of their mates [10].

The European mason bee, *Osmia cornuta* (L.), is a solitary wild and managed bee species that is an efficient pollinator of various rosaceous fruit plants [37]. Following K selection theory, *O*. *cornuta* females invest in a limited amount of high quality offspring [38–42]. The genus *Osmia* is protandrous, wherein males emerge from their cocoons a few days before their female counterparts [43]. While they wait for receptive females at nesting sites and flowers [44], they feed on floral nectar and pollen [45]. The initial days of adulthood are important for protandrous species because by locating and establishing mating sites, males are likely to increase their mating changes with females [44]. The reproductive behavior of *Osmia* species consists of three phases: courtship, copulation and post-copulatory display [30]. During the process of copulation, the ejaculate of male *Osmia* spp. coagulates in the females’ vagina, forming a so called mating plug [46]. The mating plug itself does not guarantee that further males will be prevented from mating with the female. However, it does prevent the mixing of the ejaculate and thus promotes that sperm from the first male reaches the spermatheca first [46], as females are occasionally known to mate an additional time if males fail to perform the post-copulatory display [30]. Males that are capable of copulating pass their sperm to the female spermatheca, where it remains stored for several weeks [47]. Our current understanding of *Osmia* sperm traits comes from investigations of the basic anatomy of genitalia and sperm [46] and of insemination rates and sperm counts in female spermathecae [48,49]. To our knowledge, no data exist concerning *Osmia* male sexual reproductive capacities (i.e. sperm quantity and quality) directly measured in male individuals, and how they relate to the reproductive strategy of this species.

Experimental conditions may have substantial effects on various physiological and behavioral traits [50]. Whilst laboratory studies have the advantage of a controlled environment, they may not reflect possible influences of other confounding factors (e.g. temperature, nutrition, or behavior) on a given measured parameter. For instance, poor nutritional conditions (i.e. insufficient quantity and quality of protein content and other nutrients) during larval development negatively influence body weight and over-wintering survival in *Osmia* spp. [51,52]. Other natural conditions, for instance flight behavior in bees, are equally not well represented in laboratory cage studies despite their known relevance for specific developmental procedures [53,54]. Therefore it is extremely important to establish physiological baseline information for model species under both laboratory and field conditions to better understand their biology.

Here, we quantify for the first time male reproductive traits (i.e. sperm quantity and quality) of a solitary bee using *O*. *cornuta* as a model system. Sperm traits and survival of individual solitary male bees were investigated and compared under both laboratory cage and semi-field conditions because the environment (laboratory vs. natural field conditions) may have substantial effects on measured parameters [50,55]. We predict that: (i) sperm quantity and viability of the studied probably monandrous bee species is lower compared to polyandrous ones due to lack of sperm competition in monandrous species [5], (ii) sperm quantity and viability immediately and four days post emergence differ due to the nature of protandry [56], (iii) sperm quantity and viability are positively correlated with body mass as previously shown in honeybees [19], and (iv) sperm quantity and viability of males maintained in semi-field arenas are significantly higher than of those maintained under laboratory conditions due to more natural conditions [50].

## Methods

### Experimental set-up

The study was performed in Bern and Zürich, Switzerland between April—May 2016 using European orchard bees, *Osmia cornuta*, purchased from WAB–Mauerbienenzucht, Konstanz, Germany (http://mauerbienen.com/) as cocoon-encased adults (N = 191). To prevent precocious emergence, cocoons were maintained at 2°C [57]. Immediately prior to the experiment, each cocoon was placed into a glass vial [16x2 cm] (HUBERLAB). Each vial was sealed using a cotton ball to allow air-flow, and then maintained at 20°C under complete darkness to promote adult emergence [58]. Cocoons were observed hourly to determine emergence time, defined as the period between the start of 20°C incubation and complete emergence from the cocoon [58]. Immediately following emergence, each individual was sexed [59] and visually examined to identify possible clinical symptoms of disease, parasite infestations or other abnormalities [60,61], and weighed to the nearest 0.1 mg using an analytic scale (Mettler Toledo AT400).

**Fig 1:**
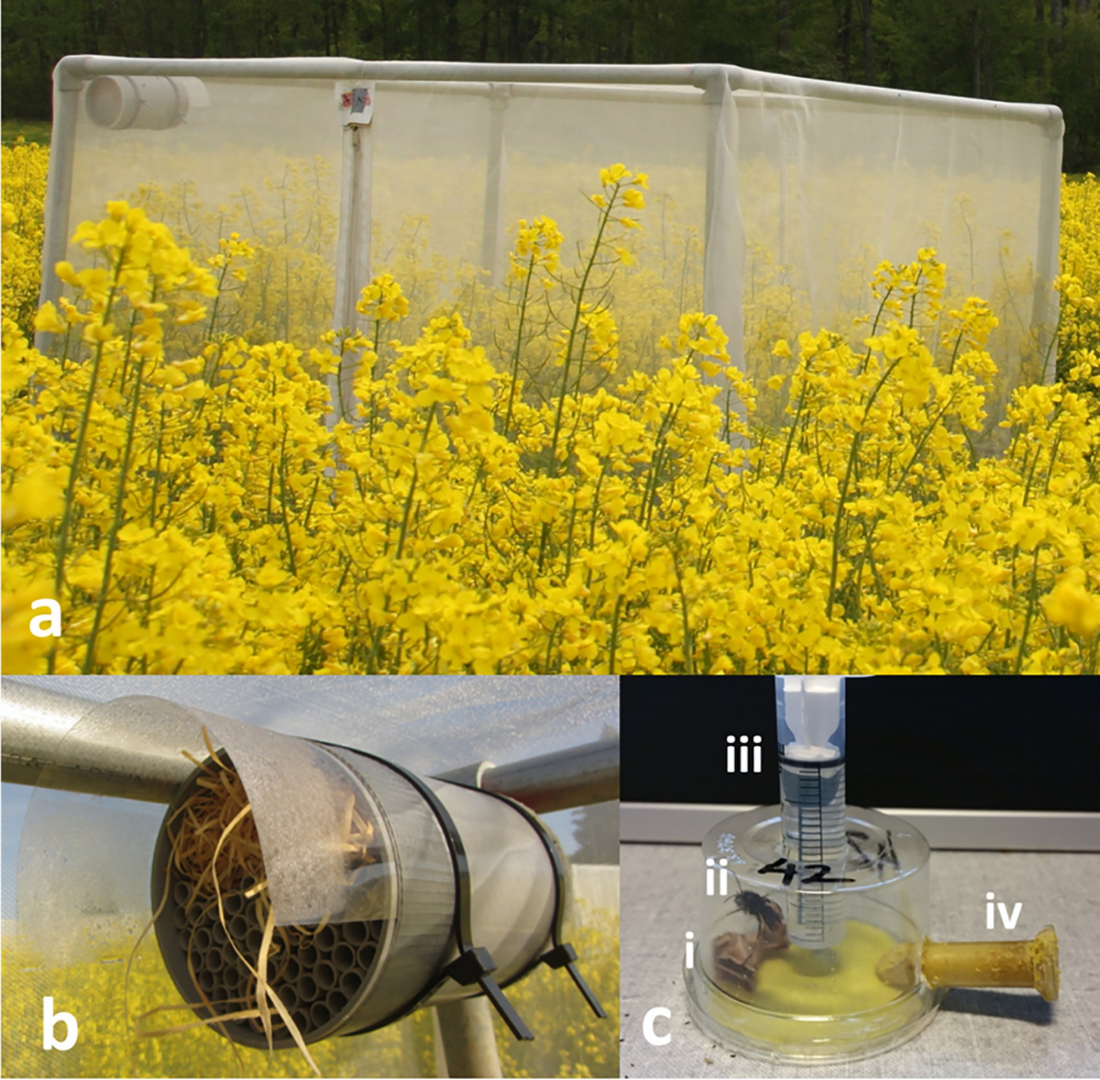
Experimental semi-field and laboratory arena set-up. **(a)** One of twelve experimental semi-field arenas [2 x 1.5 x 1.5 m] on an oilseed rape (*Brassica napus*) field. Each semi-field arena contained three male *O*. *cornuta* bees. The nesting provision is visible in the upper left-hand corner of the arena. **(b)** Each semi-field arena was equipped with a standardized nesting provision composed of a large plastic tube containing 30 paper nesting tubes (150 mm, ∅ = 9 mm, WAB–Mauerbienenzucht, Germany). **(c)** Individual *O*. *cornuta* males were placed in a standard laboratory arena [80 cm^3^] maintained at constant room temperature (24°C), with indirect natural light from a nearby window. Each arena contained: **(i)** a small piece of crumpled craft paper [2 x 2 cm] placed inside to provide a refuge, **(ii)** one male *O*. *cornuta*, **(iii)** a syringe filled with 50% (w/v) sucrose solution, and **(iv)** a modified Eppendorf tube containing pollen paste (60% fresh honeybee corbicular pollen and 40% sugar powder); *ad libitum* food supplies were replaced every 24 h to prevent possible fungus contamination.

Only males emerging within the first 24 hours and free of abnormalities and parasitism (N = 106) were randomly allocated to one of three experimental groups: 1. Immediate sperm assessment of newly emerged males (= *T*_0_, N = 34), 2. Laboratory arenas (= Laboratory, N = 36), or 3. Semi-field arenas (= Semi-field, N = 36). Each laboratory arena [80 cm^3^] [53] was maintained at room temperature (24°C) with indirect natural light [47] and contained one adult male individual (Fig 1C). Each arena was equipped with a syringe (5 ml Braun Inject) containing 50% (w/v) sucrose solution and a modified 1.5 ml Eppendorf tube containing pollen paste (60% fresh honeybee corbicular pollen and 40% sugar powder). Both food sources were fed *ad libitum* to provide adequate nutrition required for tissue and organ development [62,63]. A small piece [2 x 2 cm] of crumbled craft paper was included in each arena to provide a haven for rest and protection. Additionally, 12 field flight arenas consisting of metal piping and insect screen [1.5 x 1.5 x 2 m] (Howitec Netting BV) were set on a blooming oilseed rape (*Brassica napus*) field near Zürich, Switzerland (Fig 1A) that did not receive any pesticide applications. Each field flight arena maintained three randomly allocated males, and was equipped with an artificial nest composed of 30 standard mason bee paper straws (9 mm diameter, 150 mm length) within a plastic tube (Fig 1B) to provide a refuge. Each male was marked on the thorax with one of three unique acrylic colors (yellow, white or red) before released into the arena to allow for identification. To prevent possible bias caused by color, each individual maintained in the laboratory arenas was also marked.

### Survival and body mass assessment

Survival was assessed for individuals maintained in both the laboratory and semi-field arenas 96 hours after initial deposition into their respective experimental arenas. This is the typical time when adult males of this protandrous species first encounter receptive females [56]. Surviving individuals from both laboratory and semi-field arena conditions were then carefully removed from their respective arenas and weighed to the nearest 0.1 mg on an analytic scale (Mettler Toledo AT400) to determine post-arena body mass.

### Sperm assessment

Sperm quantity and viability were assessed using *T*_0_ and 96 hours post-experimental arena initiation (Laboratory and Semi-field individuals) individuals. Bees were briefly anaesthetized using CO_2_ before being pinned to a wax plate for dissection. Following Seidelmann (2015), the entire male genitalia consisting of the granular gland, accessary gland, seminal vesicles and testis were removed, placed in a 1.5 ml Eppendorf tube containing 200 μl Kiev^+^ buffer, and gently crushed to form a diluted stock sperm solution. Then, a 50 μl aliquot of the stock sperm solution was set aside in a separate 1.5 ml Eppendorf tube for analyses of sperm viability (proportion of living to dead sperm).

Sperm viability was quantified using the method described by Collins and Donoghue and Stürup [21,28]. In brief, each sample was diluted with 50 μl of Kiev^+^ buffer before 2 μl propidium iodide (PI) solution (1 mg ml^-1^) and 1 μl of Hoechst 33342 (0.5 mg ml^-1^) [64] (both Sigma-Aldrich) were added. Samples were incubated for ~20 min in complete darkness and then gently vortexed. Ten μl were viewed at 400x magnification using a fluorescent microscope (Olympus BX41, Switzerland) equipped with filter cubes for UV excitation [64]. Ten visual fields were selected for each sample so that quantity of living and dead sperm could be counted; an average value was calculated from these fields [64]. Sperm counts were performed by adding 50 μl of stock sperm solution diluted in 50 μl Kiev^+^ buffer (1:1 dilution) in a 1.5 ml Eppendorf tube [21,28].

Sperm quantity was measured using a Neubauer counting chamber and light microscopy (Thermo Fischer Scientific, USA) at 400x magnification. The final sperm quantity was calculated by applying the following equation [20]: sperm quantity (200 ml) = average number of sperm counted in two Neubauer counting chambers x dilution factor (1:1) x sperm volume used for Neubauer counting chamber (10 μl) x stock solution volume (200 μl). Once both total sperm quantity and sperm viability were assessed, total living sperm quantity was calculated by multiplying the two together following [20]

### Statistical analyses

All statistical tests and figures were performed using STATA15 [65]. Data were tested for normality using Shapiro-Wilk’s test for normality and visual comparisons of the data were made using Q-Q-plots. Normality tests revealed that all data were non-parametrically distributed (Shapiro-Wilk’s tests, *p* < 0.05). Therefore, non-parametric tests were used. A χ^2^- test was used to test for significant differences between the mortality rates of males in laboratory and semi-field arenas 96 hours post-arena assay initiation. Two-level generalized regression mixed models with random intercepts were fitted to analyze sperm traits. Experimental group (factor with three levels: *T*_0_, Laboratory and Semi-field) was included as a fixed term (explanatory variables), and arena ID as a random effect (because of clustering of individual bees in the semi-field arenas [66]). Likelihood ratio tests (LRT) were used to compare every two-level model with its single-level model counterpart [67]. LRTs, which did not rely on the assumption of asymptotic normal sampling distributions, were used to demonstrate which model best fit the data. Multiple pairwise comparisons (Bonferroni Test) among factor levels were obtained by using the mcompare(bonferroni) function [67]. Sperm quantity and total living sperm quantity were collected as count data and were fitted to a negative binomial model using the menbreg function. In contrast, sperm viability was scored between 0 to 100% and was analyzed using an ordered logistic model with binomial errors [68]. Lastly, XY scatter plots and Spearman’s correlation coefficient were used to assess possible relationships among sperm quantity and body mass.

Median differences and their 95% confidence intervals (CI) were calculated using the STATA15 package somersd. The function cendif calculates CI for Hodges-Lehmann median differences amongst groups [69].

## Results

An overview of all descriptive statistics regarding cocoon measurements, body mass assessments and sperm assessments are given in the S1 Table.

### Survival and body mass

**Fig 2:**
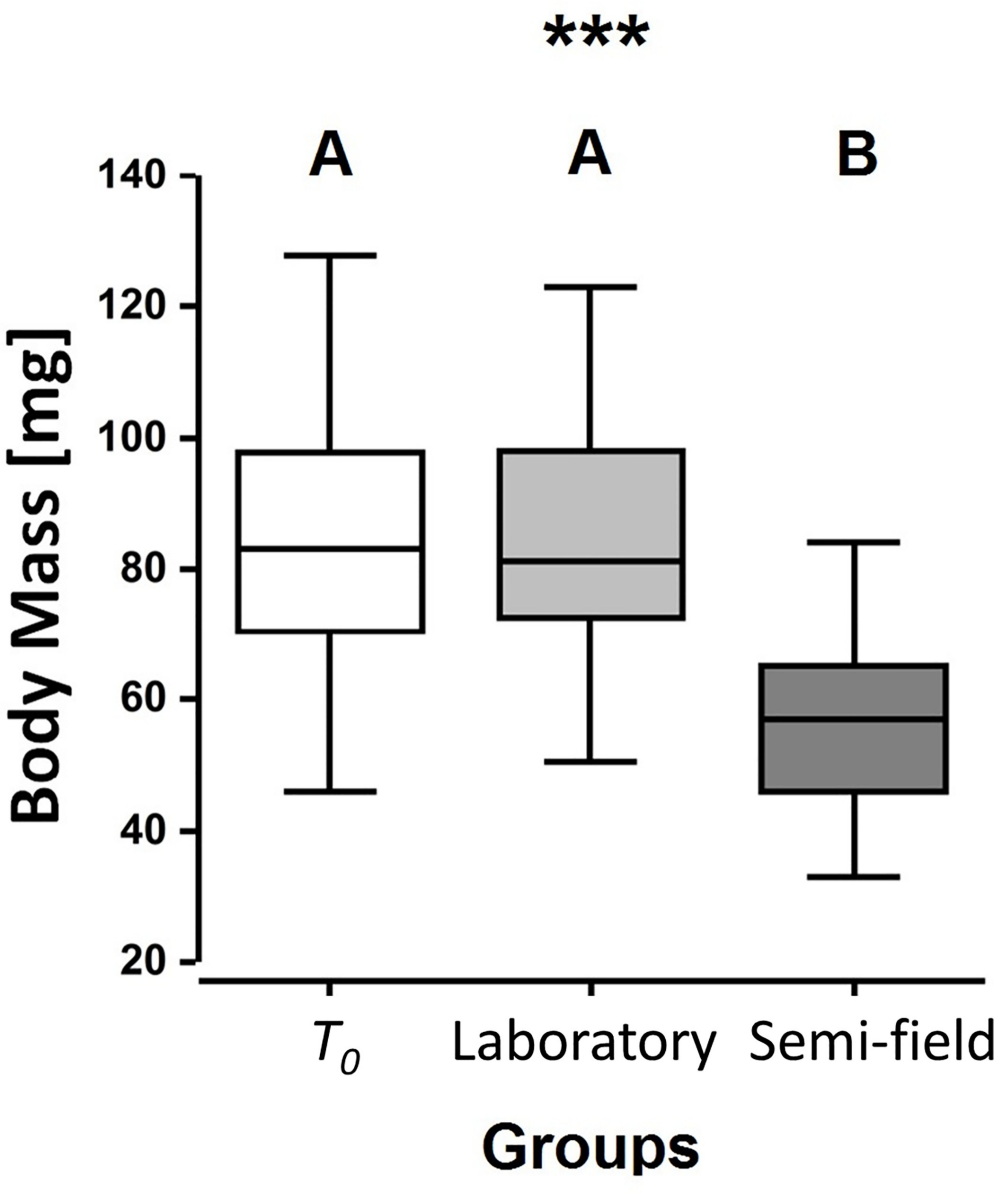
Body mass of *Osmia cornuta* males: Immediately post emergence (*T*_0_ (N = 72)), after four days under laboratory conditions (Laboratory (N = 36)) and after four days under semi-field conditions (Semi-field (N = 36)). Significant differences among groups are indicated by different letters (A, B), whereby *** represents p < 0.001.

Seventy-two males (36 per group) were used to assess the potential effects of laboratory and semi-field arenas on male survival and sperm traits. No significant difference was observed in male survival rate 96 hours post-arena assay initiation (χ^2^ = 1.06, df = 1, *p* = 0.305, S1 Fig). Laboratory and semi-field bee survival rates were 97.2% and 91.7%, respectively; however, individuals from the semi-field conditions exhibited a significantly greater loss of mass than those from the laboratory when extracted from arenas 96 hours post-arena initiation (Bmtc, all *p-*values < 0.001; Fig 2). Males from the laboratory lost 11.3 ± 41.12 - -31.34 mg, whereas males from the semi-field lost 25.5 ± 5.24- -45.86 mg (median ± 95% CI). These findings represent a relative body mass reduction of ~15% and 30% for individuals maintained in the laboratory and the semi-field, respectively.

### Sperm traits

**Fig 3:**
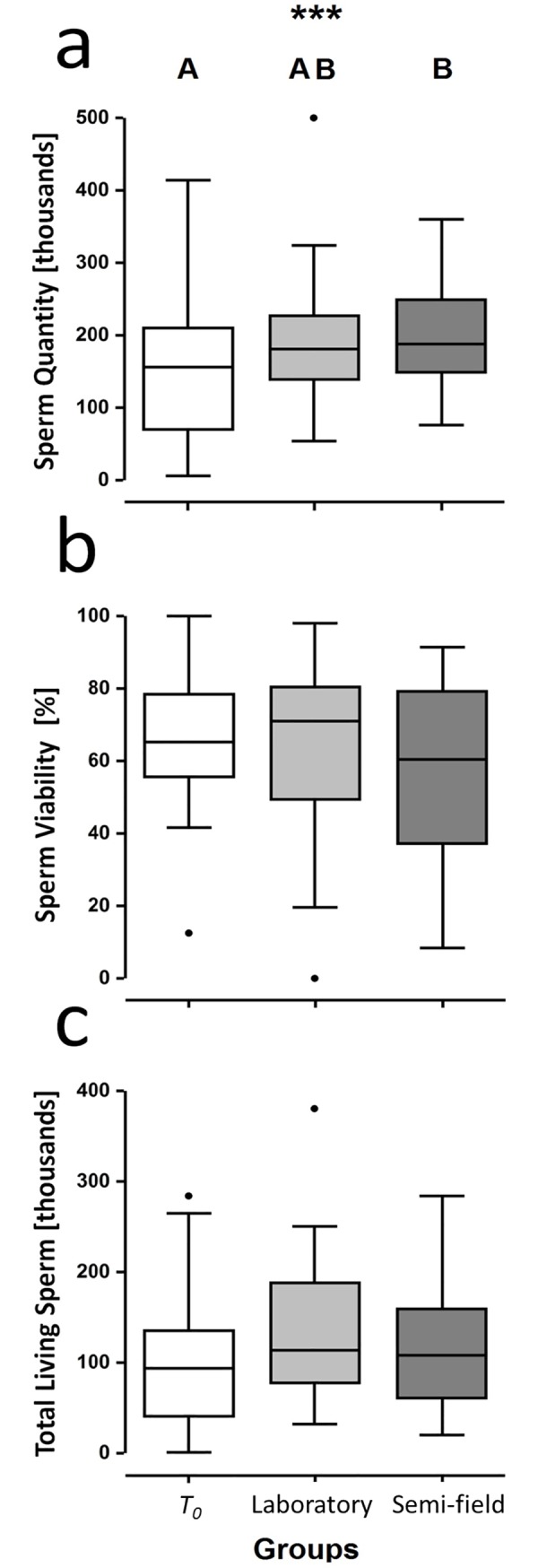
**Sperm traits of male *Osmia cornuta*: (a)** sperm quantity, **(b)** percentage of viable and **(c)** quantity of living sperm immediately post emergence (*T*_0_), four days post laboratory conditions (Laboratory) and four days post semi-field conditions (Semi-field). Significant differences among groups (p < 0.001) are indicated by different letters (A, B).

**Fig 4:**
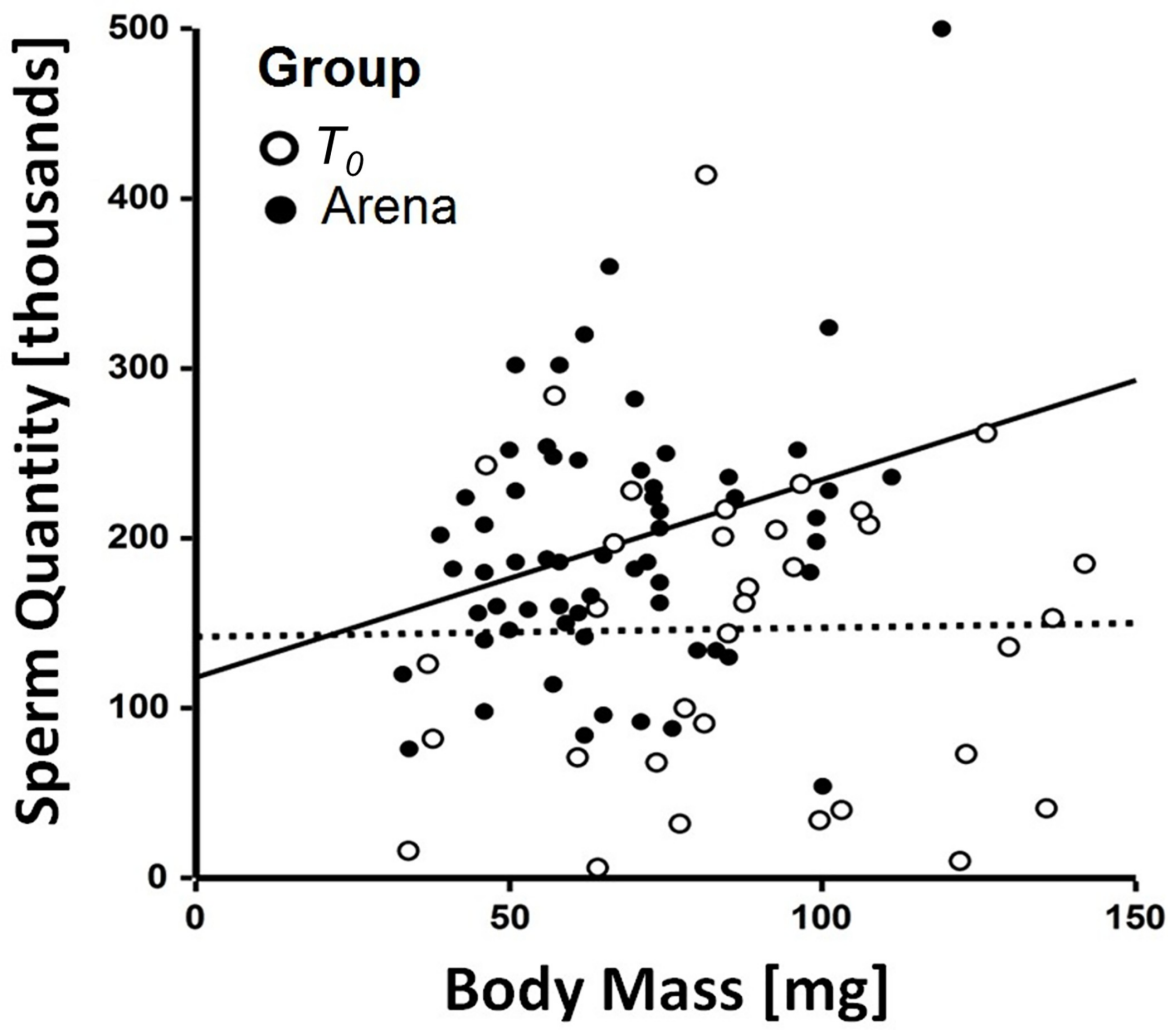
Correlation of body mass and sperm quantity in male *Osmia cornuta*: Immediately post emergence (*T*_0_) (white circles, no significant correlation between body mass and sperm quantity) and four days after emergence (black circles, solid line illustrating a significantly positive correlation between body mass and sperm quantity (Spearman’s r = 0.30).

*T*_0_ males had ~15% less sperm (median ± 95% CI: 156 ± 1–284 thousand) than semi-field males (188 ± 88–320 thousand; Bmtc, *p* < 0.001; Fig 3A). Sperm quantities in laboratory males (181 ± 84–324 thousand) were intermediate and did not significantly differ from *T*_0_ or semi-field groups (Bmtc, *p-values* = 1.0; Fig 3A). In contrast, no evidence of treatment group effects were found among *T*_0_ (65.11 ± 4.06–89.11%), laboratory (71.01 ± 19.66–92.30%) and semi-field (60.46 ± 29.10–87.97%) male sperm viability (LRT *p* = 0.74, Fig 3B). Lastly, no significant difference was observed among groups regarding total living sperm quantity (LRT, *p* = 0.24, Fig 3C). The observed median total living sperm quantities for *T*_0_, laboratory and semi-field males were 94 ± 43–265 thousand, 109 ± 32–251 thousand, 107 ± 35–282 thousand, respectively (median ± 95% CI). No significant correlation was observed between sperm quantity and sperm viability (|r| (92) = 0.10, *p* = 0.33). Body mass of four day old males (post arena, individuals from both semi-field and laboratory arenas combined) and sperm quantity were positively correlated (|r| (59) = 0.30, *p°* =°0.017, Fig 4). However, no significant relationship was observed between immediately post-emergence body mass (*T*_0_) and sperm quantity (r (32) = 0.175, *p* = 0.92, Fig 4). Body mass of newly emerged males and four day old males (combined individuals from both semi-field and laboratory arenas) did not significantly correlate with sperm viability (|r|°(32)° =°0.05, *p* = 0.77 and |r| (59) = 0.13, *p* = 0.33 respectively).

## Discussion

Our study demonstrates for the first time that the number of spermatozoa and their viability in solitary bees *O*. *cornuta* are considerably lower compared to eusocial honeybees and bumblebees [19–22,27,70]. This suggests a reduced investment in sperm by *O*. *cornuta* males, which may be linked to its life-history and/or mating system. Sperm viability immediately after adult emergence showed no significant difference compared to four day old individuals from both the laboratory and semi-field arenas, suggesting that *O*. *cornuta* males are sexually mature and capable of mating immediately post emergence. However, sperm counts were significantly higher in four day old individuals from the semi-field arena when compared to newly emerged males; this might reflect a final phase of sperm maturation [71,72]. Even though individuals from the semi-field conditions exhibited a significant loss of body mass, experimental arena had no further significant effect on any of the investigated parameters, suggesting that the given environmental conditions had no major impact.

Even though male bees may be more sensitive in laboratory trials than females [73,74], there were no significant differences in mortality rates and sperm traits between the laboratory and semi-field arenas. This suggests that under the given conditions, the environment had no significant effect. Therefore, the laboratory design employed during our study appears to provide robust estimates for future studies on solitary bees. The significant greater loss of body mass for males maintained in semi-field (30% reduction) compared to laboratory arenas (15% reduction) could be due to differences in flight activity and metabolic rates, as well as food availability and consumption rates (see *Apis mellifera* [75]).

Similar to honeybees *Apis mellifera* and stingless bees *Melipona beecheii* [19,76], the data show a significant positive correlation between body mass and sperm quantity in four day old *O*. *cornuta* males. In honeybees, increased body size may be advantageous for male-male competition [76]. In solitary bees such as *Anthidium manicatum*, body size is positively correlated with quality of male territories and mating chances [77]. The correlation between body mass and sperm counts is known in insects [78,79], and may result from different rearing environments. For example, in *A*. *mellifera* the observed correlation results from distinct brood cell types [19]. In mass provisioning solitary bees such as *O*. *cornuta*, the food given to the male offspring solely depends on the mother [80]. Since provision mass governs body size in *O*. *cornuta* [81], and larger males produce more sperm, the observed variation in sperm quantity may reflect a tradeoff scenario in female investment [81,82]. Even though no mating advantage of larger males has been reported in *O*. *rufa* (syn. *bicornis*) [44], larger males of *O*. *cornuta* may nevertheless have enhanced reproductive chances because they can inseminate more females. Indeed, multiple matings of males with up to seven females have been reported in *Osmia* [46]. Assuming similar size and filling of the *O*. *cornuta* spermatheca compared to *O*. *bicornis* (i.e. 4’000 sperm [83]), as well as the same efficacy of the sperm transfer from the oviducts to the spermatheca as in honeybees (10% efficacy)[84,85], the predicted average number of possible matings by *O*. *cornuta* males is about 4 and the maximum 12 (mean sperm number 175’000, maximum 500’000). Therefore, it can be expected that copulating *O*. *cornuta* males only release a fraction of their total ejaculate.

When comparing our data on the solitary, probably monandrous, *O*. *cornuta* bee with other bee species [10], it appears as if both the mating system (monandry vs. polyandry) as well as the level of sociality (solitary vs. eusocial) and life history may have a profound impact on the evolution of sperm quantity and quality [29,86–89]. Indeed, the range of sperm viability in *O*. *cornuta* males (60–71%) is clearly lower than in male eusocial bees (e.g. honeybee drones, >90%; bumblebee males, ~97%; S2 Table) [27,28]. Moreover, *O*. *cornuta* males produced on average 175’000 spermatozoa, which is orders of magnitude lower compared to honeybees (*A*. *mellifera*; 2.3 x 10^6^–30.3 x 10^6^ spermatozoa [19–22]). Nevertheless, honeybee queens require multiple matings to secure the complete filling of their spermatheca to ensure large and long-lived colonies due to inefficacy of the sperm transfer mechanism [90].On the other hand, sperm numbers for *O*. *cornuta* are only slightly lower than in bumblebees (*B*. *terrestris*; 230’000–500’000 spermatozoa [70,72]), however bumblebees display a higher sperm viability. Additional research is needed across a range of bee species to further advance our understanding of the role of mating systems driving male bee reproductive traits. Sperm quantity and quality interface could possibly reflect size and longevity of colonies (annual vs. perennial) in social insects. A comparative study of seven closely related insect species pairs revealed that the proportion of living sperm was consistently greater in males of polyandrous species [86]. Sperm quality plays an essential role in determining which male has an advantage when multiple males compete for fertilization [91]. The observed low sperm viability in *O*. *cornuta* males (~65%) therefore not only points into the direction of monandry, but may also offer a mechanism for the observed 6.6% failure of egg fertilization in the closely related species *O*. *bicornis* due to unsuccessful egg fertilization [92]. Regardless, reproduction of *Osmia* females is limited by the number of oocytes (40–50 [59,93]) and resource availability and the capacity for cell provisioning [42]. Accordingly, female *O*. *cornuta* lay roughly 30 eggs during their lifetime [94–98], whereby only 40% are fertilized because males are usually haploid in the hymenoptera [99]. Therefore, our data on sperm quantity and quality appears to be adaptive in light of the life history of this bee because males have to invest less compared to other species.

Our data show that males of *O*. *cornuta* are sexually mature and capable of mating with receptive females immediately post emergence similar to the closely related species *O*. *bicornis* [44]. Indeed, sperm quality of *O*. *cornuta* males does not change significantly within the first four days of adulthood. However, newly emerged males revealed a 15% lower sperm quantity when compared to four day old ones from the semi-field arenas, but not in laboratory cages. It therefore appears as if *O*. *cornuta* males also rely at least partly on a phase of sexual maturation similar to *B*. *terrestris* and *A*. *mellifera* (six and 14 days, respectively [71,72]). Since spermatogenesis in the Apidae is completed by the time of adult emergence [22,100,101], and all *O*. *cornuta* males had identical pre-emergence conditions, flight activity ([54,102]) as well as food quantity and quality [76,103,104] may explain the observed age specific differences in sperm quantity.

## Conclusions

Our novel findings on low sperm quantity and viability in a solitary bee support the idea that sperm traits in bees may have evolved according to the mating strategy (i.e. monandrous vs. polyandrous), as well as life history and degree of eusociality (e.g. solitary vs. eusocial). Moreover, sperm traits can be important proxies in evaluating environmental hazards [20] and therefore a solid understanding of sperm maturation and time of sexual maturity in males of solitary wild bees appears crucial from a conservation point of view. However, additional studies in more bee species with different mating strategies i.e. known polyandrous ones (e.g. within the family of Megachilidae and Andrenidae [105,106]), and life histories across varying environments, are required before being able to derive general conclusions.

## Supporting information

S1 Fig Adult survival four days post arena exposure in male *Osmia cornuta*: Survival was assessed for individuals maintained under laboratory (Laboratory (N = 36)) and semi-field (Semi-field (N = 36)) arenas four days after arena assessment initiation. No significant difference was observed between male *O*. *cornuta* bee maintained under laboratory and semi-field arena conditions (Chi-square test, χ^2^ = 1.06, df = 1, *p* = 0.305).
(TIF)

S1 Table Summary of descriptive results for all measured parameters for both female and male *Osmia cornuta*. (XLSX)

S2 Table Overview of sperm traits from various bee species in relation to their mating strategies and eusociality. Not available data is represented as N.A.
(XLSX)
