# Supplementary figures and images for: Not every sperm counts: Male fertility in solitary bees, Osmia cornuta
Source: PLoS One. 2019 Mar 28;14(3):e0214597. doi: 10.1371/journal.pone.0214597 (PMC6440592; doi:10.1371/journal.pone.0214597)

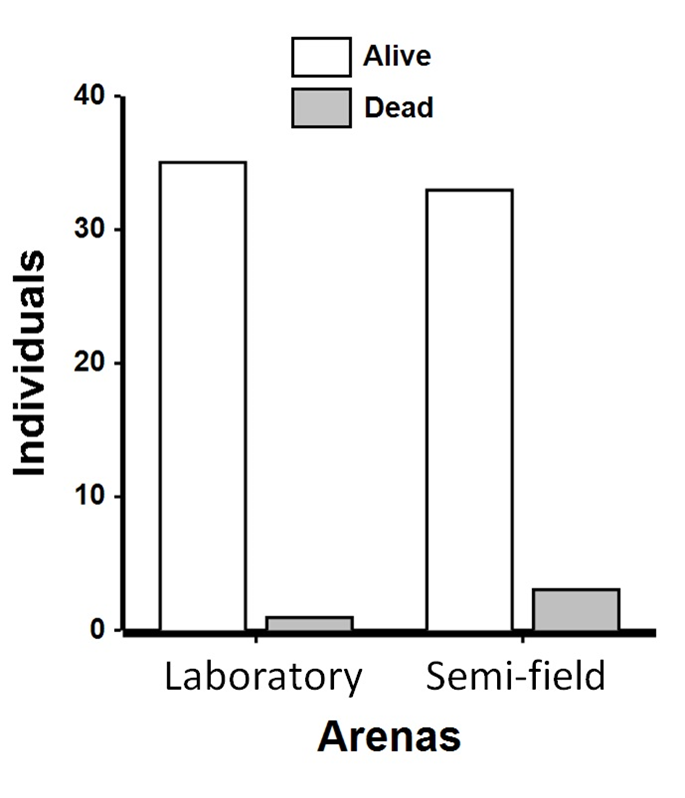

Supplement: S1 Fig — No significant difference was observed between male O. cornuta bee maintained under laboratory and semi-field arena conditions (Chi-square test, χ2 = 1.06, df = 1, p = 0.305). (TIF) [file pone.0214597.s001.tif]
